# Clinical Relevance of Telomere Status and Telomerase Activity in Colorectal Cancer

**DOI:** 10.1371/journal.pone.0149626

**Published:** 2016-02-25

**Authors:** Tamara Fernández-Marcelo, Andrés Sánchez-Pernaute, Irene Pascua, Carmen De Juan, Jacqueline Head, Antonio-José Torres-García, Pilar Iniesta

**Affiliations:** 1 Department of Biochemistry and Molecular Biology II, Faculty of Pharmacy, Complutense University, 28040-Madrid, Spain; 2 Service of General Surgery and Digestive Tract, San Carlos Hospital, 28040-Madrid, Spain; 3 Sanitary Research Institute of San Carlos Hospital (IdISSC), 28040-Madrid, Spain; University of Nebraska Medical Center, UNITED STATES

## Abstract

The role of telomeres and telomerase in colorectal cancer (CRC) is well established as the major driving force in generating chromosomal instability. However, their potential as prognostic markers remains unclear. We investigated the outcome implications of telomeres and telomerase in this tumour type. We considered telomere length (TL), ratio of telomere length in cancer to non-cancer tissue (T/N ratio), telomerase activity and *TERT* levels; their relation with clinical variables and their role as prognostic markers. We analyzed 132 CRCs and paired non-cancer tissues. Kaplan-Meier curves for disease-free survival were calculated for TL, T/N ratio, telomerase activity and *TERT* levels. Overall, tumours had shorter telomeres than non-tumour tissues (P < 0.001) and more than 80% of CRCs displayed telomerase activity. Telomere lengths of non-tumour tissues and CRCs were positively correlated (P < 0.001). Considering telomere status and clinical variables, the lowest degree of telomere shortening was shown by tumours located in the rectum (P = 0.021). Regarding prognosis studies, patients with tumours showing a mean TL < 6.35 Kb experienced a significantly better clinical evolution (P < 0.001) and none of them with the highest degree of tumour telomere shortening relapsed during the follow-up period (P = 0.043). The mean TL in CRCs emerged as an independent prognostic factor in the Cox analysis (P = 0.017). Telomerase-positive activity was identified as a marker that confers a trend toward a poor prognosis. In CRC, our results support the use of telomere status as an independent prognostic factor. Telomere status may contribute to explaining the different molecular identities of this tumour type.

## Introduction

Colorectal cancer (CRC) is the third most common cancer in men and the second in women worldwide[1]. Several factors are associated with a lower CRC probability, such as high physical activity and high intakes of dietary fiber, fish, nuts, dairy products, and fruits and vegetables. However, high body mass index and waist circumference, smoking, alcohol consumption, and red and processed meat intakes are related to a higher CRC risk [2–4].

Different genetic and epigenetic events are involved in the progression of CRC [5]. Currently, three pathways, not mutually exclusive and named by the cellular process which promotes them, have been implicated in the development of CRC. The chromosomal instability (CIN) pathway is characterized by the accumulation of numerical or structural chromosomal abnormalities which causes the loss of onco-suppressor genes and the activation of oncogenes. The microsatellite instability (MSI) pathway is defined as a deficient DNA Mismatch Repair System that causes unrepaired errors in the microsatellites (nucleotide repeat sequences). Finally, the CpG island methylator phenotype (CIMP) pathway exhibits gene silencing due to a widespread hypermethylation of CpG islands at several loci [6–9].

Approximately 85% of CRCs are characterized by chromosomal instability and telomere dysfunction may be considered a major driving force in generating this feature [9]. Telomeres are repetitive DNA sequences (TTAGGG) located at the end of chromosomes which are associated with the Shelterin complex. Among its main functions, telomeres protect chromosomes from end-to-end fusion and DNA degradation [10,11]. The end replication problem, owing to the inability of DNA polymerase to replicate the 3’ end of chromosomes, causes a progressive telomere shortening which is coupled to cell division. When a critical telomere length is reached, cells activate the protective mechanisms, i.e. senescence & apoptosis, in order to avoid the proliferation of genetically unstable cells. If these mechanisms fail, cells continue to divide and enter into a crisis stage which is characterized by massive genomic instability and cell death [9,12,13]. At this point, the activation of telomerase (a ribonucleoprotein complex composed of an internal RNA component and a catalytic protein with telomere-specific reverse transcriptase activity) compensates for telomere shortening and allows cells successfully exit from the crisis stage. Telomerase is specifically expressed in immortal cells, such as stem cells, germ tissues and cancer cells. For the latter, telomerase confers an unlimited replicative potential and has been implicated in immortalization and carcinogenesis [13–16].

Telomere shortening is an early event in CRC carcinogenesis and telomere/telomerase dysfunction is considered as a fundamental player in this process [9]. For telomerase, there is a general consensus that high hTERT levels or telomerase activity are associated with poor prognosis [17,18]. However, the prognostic role of telomere length in CRCs still needs to be confirmed [9]. Recently, the clinical utility of telomeres has been demonstrated to improve the treatment of metastatic colorectal cancer: the outcome of patients treated with anti-EGFR therapy seems to be dependent on the tumour telomere length [19]. While most studies identify telomere shortening as a critical initial event in carcinogenesis, the role of telomere length in cancer cells as a marker of disease progression is controversial [20]. In fact, considering CRC, mean telomere attrition has been associated with the lymph node invasiveness of colon cancer cells [21], whereas other works indicated that telomere length in cancer tissue was significantly longer in the late stage of colorectal tumours [22].

Considering the relevance of telomere and telomerase in carcinogenesis and, in particular, their role as prognostic biomarkers, our main aim in this work was to highlight and help to clarify the importance of the above mentioned parameters of telomere function as independent prognostic biomarkers in CRC. With this objective, we evaluated the telomere status (telomere length & T/N ratio) and telomerase, their relation with clinical variables, and their role as molecular biomarkers for predicting the outcome of a large series of patients with CRC operated on.

## Materials and Methods

### Patients and tissue samples

One hundred and thirty-two colorectal carcinomas (CRCs), and their corresponding control tissue samples, were obtained from patients who had undergone potentially curative surgery at San Carlos Hospital in Madrid, Spain, between 2006 and 2010. Paired normal tissues from the same patient, used as controls, were obtained and microscopically confirmed. After surgical resection, all tissue samples were snap-frozen in liquid nitrogen and stored at -80°C until processed. Cryostat sectioned, H&E stained samples from each tumour block were examined microscopically by two independent pathologists to confirm the presence of ≥ 80% tumour cells. All the cases were collected without selection in function of gender, age or tumour stage and no patient had received previous chemotherapy or radiation therapy before diagnosis and entry into this study. Informed written consent was obtained from patients prior to investigation and this study was approved by the Ethical Committee of the Hospital.

Colorectal tumours were staged pathologically as the Dukes Stage A-D, according to the modification of the original Dukes staging scheme by Turnbull el al [23]. Therefore, 17 tumours were classified as stage A, 55 as stage B, 33 as stage C and 18 as stage D. Dukes Stage was not available for 9 cases. The median follow-up period was 5 years, (range, 1–110 months).

### Telomere length and Telomerase measurement

**Fig 1:**
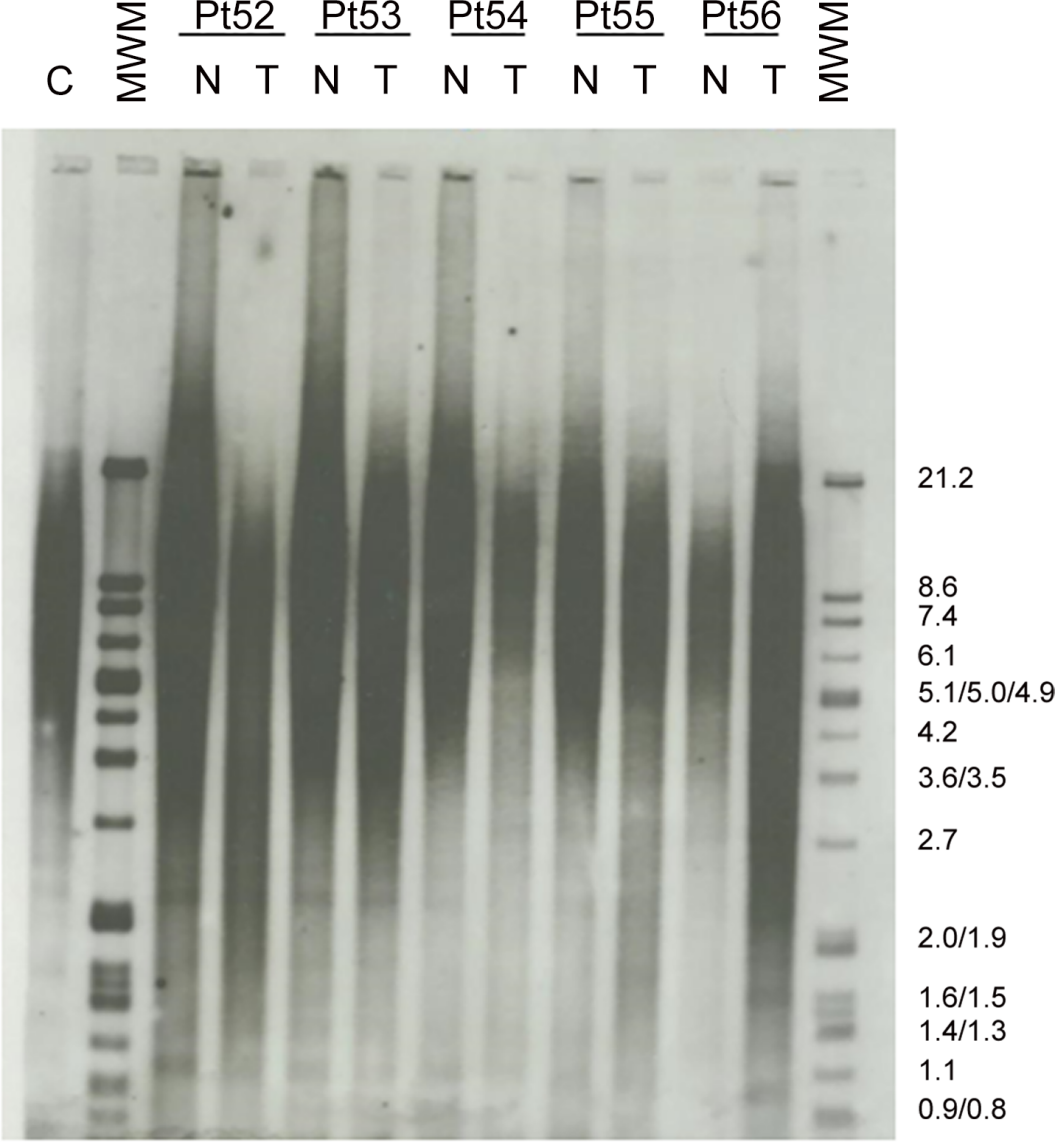
Results of a representative x-ray film for telomere length analysis from five colorectal cancer patients (Pt) in tumour tissues (T) and their paired non-tumour samples (N). C, positive control. MWM, DNA molecular weight marker.

Terminal restriction fragment (TRF) length measurement was performed using Telo TTAGGG Telomere Length Assay kit, Cat No. 12 209 136 001 (Roche Applied Science, Penzberg, Germany) as previously described [18]. TRF lengths for tumour and control tissues were determined by comparing the signals relative to a standard molecular weight using Image Gauge software version 3.46 (Fujifilm, Tokyo, Japan). The TRF length ratio was determined as the ratio of the length of tumour tissue TRF and their paired normal tissue TRF (T/N ratio). In Fig 1, a representative x-ray film for telomere length analysis can be observed. All lanes were divided into intervals and mean TRF length was defined as ∑(OD_i_)/ ∑(OD_i_/L_i_) where OD_i_ is the chemiluminescent signal at a given position (i) and L_i_ is the length of the TRF fragment at the same position (i). Shortening or lengthening of TRFs was defined if the TRF length of tumour tissues was shorter or longer than the corresponding non-tumour tissues, respectively.

In colorectal samples, telomerase was measured using the telomeric repeat amplification protocol (TRAP)-based telomerase polymerase chain reaction (PCR)-enzyme-linked immunosorbent assay (ELISA) kit Cat No. 11 854 666 910 (Roche Applied Science, Penzberg, Germany), which allowed us to establish a semiquantitative assay [24]. Thus, considering that the cut-off for TRAP-ELISA negativity corresponds to an optical density OD _450 nm_ < 0.2, all samples with OD _450 nm_ ≥ 0.2 were considered telomerase positive [25].

### Quantification of *TERT* expression

Total RNA was extracted from tumour tissues using TRIzol^®^ (Thermo Fisher Scientific, Waltham, MA USA) according to the manufacturer’s instructions. Reverse transcription reactions were performed with 2 μg of total RNA using the High Capacity cDNA reverse transcription kit (Thermo Fisher Scientific) following the manufacturer’s instructions. The expression of *TERT* was measured by real-time quantitative PCR (qRT-PCR) using the Taqman probe *TERT* (Hs99999022_m1, Thermo Fisher Scientific). The amount of target (Ct _*TERT*_) was normalized to the expression of the endogenous reference Cyclophilin A, *PPIA* (Ct _*PPIA*_) (Hs99999904_m1, Thermo Fisher Scientific) [25]. Thus, the results are expressed as ΔCt values (Ct _*TERT*_—Ct _*PPIA*_); note that lower ΔCt values equated to increased mRNA expression levels.

### Statistical analysis

Statistical analyses were performed using the SPSS software package version 19 (SPSS Inc., Chicago, IL, USA). Differences in telomere length and T/N ratio among various groups of patients, discriminated for clinical variables, were analysed by the Student-T and ANOVA tests, or their non-parametric alternatives, Mann-Whitney U test and Kruskal-Wallis test. P-value < 0.05 was considered statistically significant. The paired samples T test was used for comparing the means of two related variables or its non-parametric option, the Wilcoxon test. Chi-square test was used to determine a relationship between two categorical variables. Correlations were assessed by the Spearman test.

Group-oriented curves for disease-free survival (DFS) were calculated according to the Kaplan-Meier method considering telomere length, T/N ratio, telomerase activity and *TERT* expression. DFS was calculated from the day of surgery until recurrence. The differences in DFS across different groups were compared using the log-rank test. The relative prognostic impact of telomere length compared with established prognosis factors was evaluated by Cox univariate and multivariate analyses. The mark of censored data indicates the end of an individual follow-up period. Cutoff Finder Web Application (http://molpath.charite.de/cutoff/) [26] was used to determine the cut-off points for prognosis analysis.

## Results

### Telomere status and telomerase activity in tissue samples

Telomere length was analysed in a total of 264 colorectal tissue samples: 132 CRCs and their corresponding non-tumour samples. The mean telomere lengths were 5.49 ± 0.23 Kb in tumour tissue samples and 7.32 ± 0.33 Kb in non-tumour samples. Colorectal tumours displayed significantly shorter telomeres than non-tumour tissues (P < 0.001, Wilcoxon test). The mean T/N ratio was 0.79 ± 0.02. One hundred and four tumours (104/132; 82.5%) showed positive telomerase activity and twenty-two (22/132; 17.5%) were telomerase negative. Telomerase activity was not available for six tumours.

### Telomere status and telomerase activity: correlation with clinical variables

The study population was integrated by 132 patients with a median age of 71 years. An inverse correlation between telomere lengths of CRC samples and age was found (r = -0.176; P = 0.066, Spearman test) and the same trend for telomere lengths of non-tumours tissues and age (r = -0.256; P = 0.007, Spearman test). Moreover, telomere lengths of tumours and paired control tissues were positively correlated (r = 0.685; P < 0.001, Spearman test).

In CRC samples there was a trend to association between the telomere length and the Dukes stage (P = 0.086, Kruskal-Wallis test) (Table 1). Moreover, a significant association was found between the T/N ratio and tumour location (P = 0.021, one-way ANOVA) (Table 1): tumours located in the rectum showed the highest T/N ratio, in relation to the tumours located in the right or left colon (Table 1). No significant differences were found between the gender and tumour telomere length or T/N ratio; neither to Dukes stage and T/N ratio nor tumour location and tumour telomere length (Table 1). Finally, no associations were established between the clinical variables and the mean telomere length of non-tumour samples (Table 1). A statistical relation could not be established between gender, Dukes stage, tumour location and telomerase activity (Table 2).

**Table 1:** Telomere status and clinical variables in colorectal samples.

		Telomere length (Kilobase pairs; mean ± standard error)					
Variable	N° of cases	Tumour samples	P and test statistic	Non-tumour samples	P and test statistic	T/N ratio (mean ± standard error)	P and test statistic
Gender	132		0.685; Mann-Whitney U Test		0.418; Mann-Whitney U Test		0.803; T test
Female	70	5.41 ± 0.33		7.12 ± 0.47		0.80 ± 0.03	
Male	62	5.59 ± 0.33		7.55 ± 0.46		0.79 ± 0.03	
Dukes stage	123^#^		0.086; Kruskal-Wallis test		0.151; Kruskal-Wallis test		0.749; one-way ANOVA
A	17	4.49 ± 0.44		6.37 ± 0.66		0.73 ± 0.03	
B	55	5.19 ± 0.35		6.79 ± 0.43		0.80 ± 0.04	
C	33	6.22 ± 0.51		8.72 ± 0.90		0.79 ± 0.05	
D	18	6.11 ± 0.69		7.75 ± 0.82		0.80 ±0.04	
Tumour location	125^†^		0.288; Kruskal-Wallis test		0.476; Kruskal-Wallis test		0.021; one-way ANOVA
Right colon	34	5.01 ± 0.38		7.63 ± 0.86		0.74 ± 0.03	
Left colon	30	5.63 ± 0.63		8.14 ± 0.75		0.72 ± 0.05	
Rectum	61	5.74 ± 0.31		6.86 ± 0.35		0.85 ± 0.03	

^#^Dukes stage was not available for 9 cases.

^†^Tumour location was not available for 7 cases.

**Table 2:** Telomerase activity and clinical variables in colorectal cancer.

		Telomerase activity. N° of cases and %		
Variable	N° of cases	Negative	Positive	P; Chi-square test
Gender	126			0.823
Female	66	12 (18.2%)	54 (81.8%)	
Male	60	10 (16.7%)	50 (83.3%)	
Dukes stage	118			0.639
A	16	2 (12.5%)	14 (87.5%)	
B	51	9 (17.6%)	42 (82.4%)	
C	33	5 (15.2%)	28 (84.8%)	
D	18	5 (27.8%)	13 (72.2%)	
Tumour location	119			0.239
Right colon	31	8 (25.8%)	23 (74.2%)	
Left colon	30	3 (10%)	27 (90%)	
Rectum	58	9 (15.5%)	49 (84.5%)	

### Telomere status, telomerase activity and CRC prognosis

The prognostic value of telomere status (telomere length & T/N ratio) and telomerase activity was explored by prognosis studies in patients with resected CRCs. Only patients who had undergone potentially curative surgery (Dukes stage A, B or C) and who did not die during the postoperative period were considered in these studies, as established in the literature.

**Fig 2:**
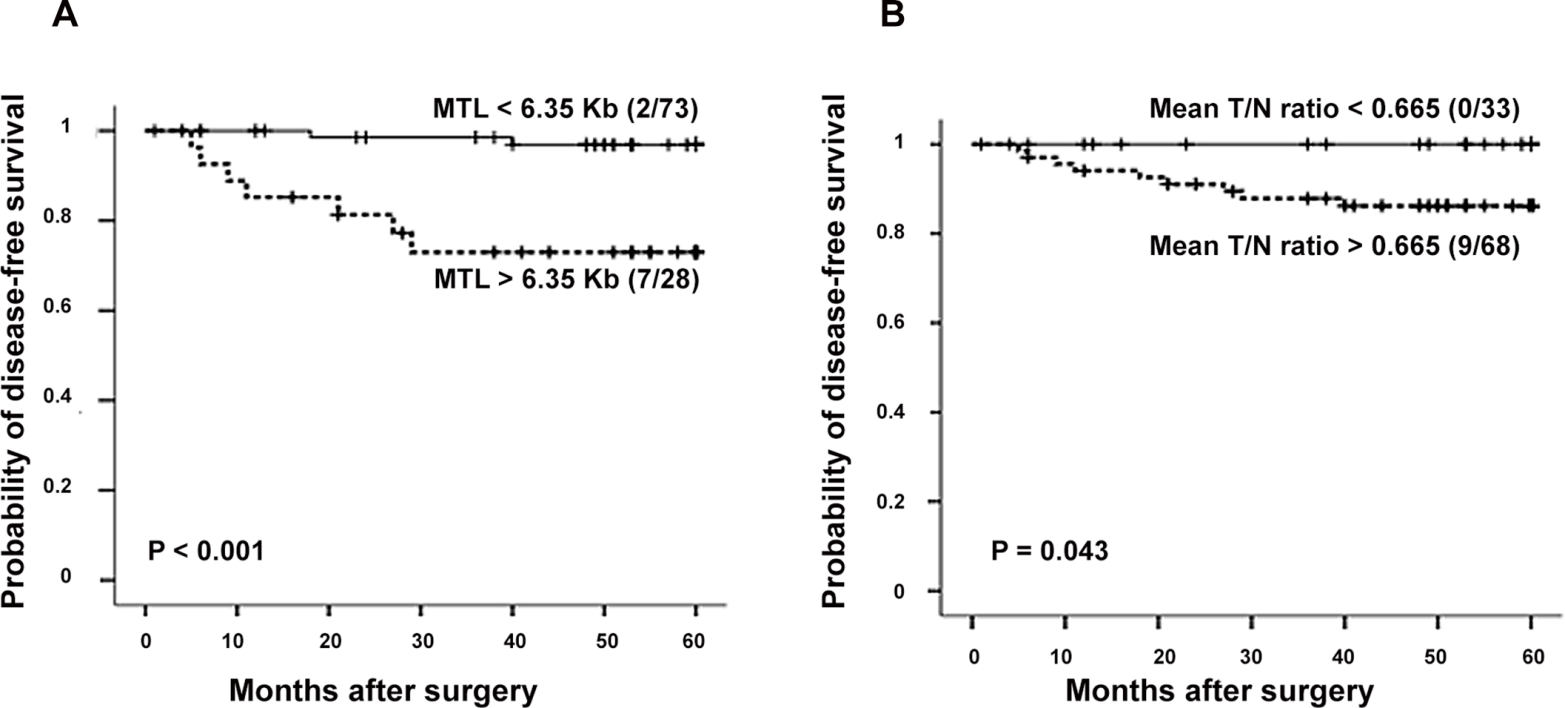
Kaplan-Meier survival curves considering telomere status in CRC population. **(A) Kaplan-Meier survival curves in relation to the mean telomere length (MTL). (B) Kaplan-Meier survival curves in relation to the T/N ratio.** Numbers in brackets represent cases with tumour recurrence and crosses indicate censored data.

The study population consisted of 101 patients for prognosis analyses considering telomere status. Optimal cut-offs for the mean tumour telomere length (6.35 Kb. P < 0.001; Log-Rank) and mean T/N ratio (0.665. P = 0.043; Log-Rank) were calculated using the Cutoff Finder Web Application (http://molpath.charite.de/cutoff/) [26]. The patients with a mean tumour telomere length < 6.35 Kb experienced a significantly better clinical evolution compared to the group of patients whose mean tumour telomere length was > 6.35 Kb (Fig 2A). Moreover, none of the patients with the highest degree of telomere shortening in the tumour relapsed during the follow-up period (Fig 2B).

Following, a Cox multivariate analysis was performed to identify which independent factors jointly had a significant influence on survival. For this study, variables with a P-value lower than 0.15 in the univariate analysis were selected (Dukes stage of CRC and mean telomere length of tumours) (Table 3). As result, the mean telomere length in colorectal tumours was shown to be an independent prognostic parameter for disease-free survival (P = 0.017) in the multivariate analysis. The risk of relapse for the 73 patients with a mean tumour telomere length < 6.35 Kb was lower compared to the 28 who had a mean tumour telomere length > 6.35 Kb (Table 3). Also, we corroborated that the prognostic implication of tumour telomere length in CRC does not vary with age. Thus, our results indicated that the prognostic value of telomere status is retained even when the median age of patients was considered. After comparing the survival data for patients with a median age < 71 years with those of patients with a median age > 71 years, tumours showing shorter telomeres (< 6.35 Kb) conferred the best clinical evolution (P < 0.001). It was not mathematically possible to establish the Cox multivariate study including the mean T/N ratio, because one subset of events was empty (no cases of recurrence within the group of mean T/N ratio < 0.665) and an undefined value for the relative risk would have been obtained.

**Table 3:** Univariate and Multivariate Cox Regression Analysis in colorectal cancer.

		Multivariate Analysis		
Variable	P (univariate analysis)	P	RR	CI
Sex, male *vs*. female	0.931	_	_	_
Age, <71 years *vs*. >71 years	0.510	_	_	_
Dukes stage, A or B *vs*. C	0.003	0.008	0.059	0.007 to 0.48
Tumour location, colon *vs*. rectum	0.280	_	_	_
Mean telomere length in CRC, <6.35 Kb *vs*. >6.35 Kb	0.003	0.017	0.145	0.03 to 0.71

RR, relative risk. CI, confidence interval.

No significant associations were found between the categories based on telomere length and telomerase activity (P = 0.165, Chi-square test) nor T/N ratio and telomerase activity (P = 0.062, Chi-square test).

Telomerase-positive activity was identified as a marker of a trend toward a poor prognosis. During the follow-up period, no recurrences were detected in the group of patients who had telomerase-negative tumours (P = 0.184, Log-Rank). Finally, an optimal cut-off (ΔCt value 11.2; P = 0.26) for the *TERT* expression was calculated using the Cutoff Finder Web Application[26]. However, no significant prognostic differences were found between the two groups defined by *TERT* expression (ΔCt value < 11.2 *vs* ΔCt value > 11.2).

## Discussion

The early diagnosis of colorectal cancer has contributed to diminishing its incidence rate. Moreover, the incorporation of new predictive and prognostic biomarkers has enabled specifying the efficacy of the therapies and the treatment response, and predicting, more accurately, the likely outcome of cancer [27]. Previous research works have evaluated the role of telomere status for identifying groups of metastatic colorectal cancer patients susceptible to benefiting from a specific therapy[19]; also, as a prognostic factor [9] and, recently, telomere status has demonstrated to be useful for sub-classifying the rectal cancer [28].

Telomere biology is complex: protecting chromosome ends from being recognized as DNA breaks, but telomeres shorten with age in a way coupled to cell division [29]. Thus, our results show a significant inverse correlation between the age of patients and telomere lengths of non-cancerous colorectal tissues, in line with previous studies [30,31]. Likewise, a decrease of telomere lengths of colorectal cancers with the age of patients was detected, although not conclusive, suggesting that tumour cells inherit the proliferative story of the healthy tissue. This fact could be reinforced by the significant and positive correlation found in this study between telomere lengths in non-cancerous colorectal tissues and tumours. The negative correlation between telomere length and age has been demonstrated in breast tumour tissues [32], but the literature is not conclusive for colorectal cancer [18,31]. For other tumour types, such as esophageal cancer, it is suggested that other factors (oxidative stress and life stress) affect telomere length [32]. In addition to the proliferative story of the healthy tissue, the strong replicative potential of cancer cells influences tumour telomere lengths: the majority of tumours have telomeres similar or shorter in length than adjacent normal tissues [33]. For colorectal cancers, it is well established that telomeres are shorter compared with adjacent mucosa [9], and our results are in line with this fact. Previous studies have demonstrated an increased telomerase activity in colorectal cancer tissues [18,34]. In fact, 90% of human tumours maintain telomere lengths through telomerase, with the aim of compensating telomere shortening and guaranteeing the proliferative capacity of cancer cells [33]. More than 80% of the tumours evaluated in this study showed telomerase activity, in agreement with previously published data [18].

The study of telomere length and telomerase activity, in relation to clinical variables, may shed light on colorectal cancer biology. Considering tumour location, the prognosis of rectal cancers is worse than that of colon cancers and the clinical treatment is different [35]. In relation to telomere length and telomerase activity, previous results demonstrated that telomere length differed according to tumour location, being longer in rectal cancers [15]; it has been recently demonstrated that telomerase activity is lower in rectal cancer as compared to colon cancer [36]. Although no differences were detected between the telomere lengths of colorectal cancers and non-tumour tissues and the bowel anatomy, our results suggest that the dynamic of telomere shortening is different according to tumour location, as the lowest degree of telomere shortening, i.e. the highest T/N ratio, is reached by the tumours located in the rectum. Thus, telomere status and telomerase activity may contribute to defining the identity of colon and rectal tumours and improving the therapies. Moreover, a relationship between telomere status and tumour stage was identified: the highest degree of telomere shortening and the shortest telomeres were found in tumours of Dukes stage A, in agreement with previously published data [31], supporting the hypothesis that sufficient telomere stabilization, mediated by hTERT is achieved late in tumorigenesis [37].

In colorectal cancer, the prognosis and therapy decision only based in the specific tumour stage sometimes become insufficient for predicting the treatment response and disease recurrence. So, the incorporation of new biomarkers is required. Previous research showed the prognostic role of telomere length and telomere length ratio in colorectal cancer[9], but a “healthy range” is still needed [38]. Here, we demonstrate a threshold for telomere length and telomere length ratio, which defines a differential clinical outcome: the patients with a mean tumour telomere length < 6.35 Kb experienced the best clinical evolution and none of them with the highest degree of telomere shortening suffered a recurrence during the follow up period. Thus, longer telomeres indicate poor prognosis in colorectal cancer. A possible molecular explanation for this fact could be the differential regulation of telomerase expression in colorectal epithelial cells [39]. Also, it may be possible that the viability of senescence and cell death pathways, in response to short telomeres, contributes to limiting tumorigenesis, as we previously hypothesized [40]. Our results seem to indicate that the telomere attrition could be acting as a tumour suppressor mechanism in CRC. However, considering that the inappropriate expression of telomerase, which maintains telomere length, allows the overcoming of cellular senescence and apoptosis [20], additional molecules related to telomere maintenance and genome instability should be investigated in order to completely explain the molecular basis of the favorable clinical evolution of CRC patients with telomere shortening studied in this work.

As has been previously mentioned, telomere shortening is a crucial event in carcinogenesis which has been found in different tumour types, including CRC. Also, telomeres have been considered within the context of therapy [41]. However, the use of telomeres in the clinical management of CRC is still a challenge. In that tumour type and others (e.g. gastric cancer) the results are discordant in relation to the telomere alterations and disease progression [20], so more studies are needed to clarify this issue. Our results try to strengthen the better clinical prognosis conferred by telomere shortening in CRC patients and are in line with previously published data: in a population of 57 patients with colorectal carcinoma, analysed by Dr Gertler and cols [31], a significantly higher overall survival rate was shown in the group of patients with a T/N ratio ≤ 0.9. Moreover, the higher overall survival rate was displayed by the group of CRC patients analysed by Dr Valls and cols [42] with a T/N ratio ≤ 1 and negative lymph node involvement. Since there is no agreement concerning the role of telomeres as marker of disease progression in CRC, it is quite important to compile results in order to reach a consensus. Regarding our study, we can underline the large number of patients considered in the prognosis analysis as well as the use of the Cutoff Finder Web Application [26], which allowed us an objective distinction between groups with a different clinical prognosis based on the telomere status.

Moreover, we have demonstrated in the current work the potential clinical use of telomerase activity for predicting colorectal cancer recurrences. In relation to the prognosis of CRC patients, our data only showed a trend toward a worse clinical evolution in those with telomerase positive tumours; and, when *TERT* levels are considered, no significant prognostic differences were found between the two groups of CRC patients defined by *TERT* expression. However, in other studies, telomerase had been identified as an independent prognostic marker for overall survival in colorectal cancer patients: patients with high *TERT* levels showed a significantly worse overall survival than those with lower *TERT* levels [17]. The consensus has not been reached [9] and further studies are needed to ascertain the cut-off value which distinguishes between the populations with different prognosis. Quantification of *TERT* mRNA and quantification of telomerase activity are the two main strategies employed to estimate telomerase levels [9]; moreover, telomerase activity estimated by TRAP correlates with the levels of *hTERT* mRNA, as we previously published [25]. Therefore, the dichotomization of cancer population considering telomerase activity could be useful for identifying groups of patients susceptible to receiving therapies aimed at the inhibition of telomerase.

## Conclusion

In colorectal cancer, our results support the use of telomere status (telomere length & T/N ratio) as an independent prognostic factor for predicting the clinical outcome. The novel finding of this study is the independent prognosis role of a specific telomere status in CRC patients. Telomere function may contribute to explaining the different molecular identities of this tumour type. Colorectal cancer is still an important cause of cancer related deaths; thus, the consideration of new biomarkers for anticipating recurrences and helping in therapy decisions may provide an improvement in the clinical management of this disease.
